# Waste Glass Powder Reusability in High-Performance Concrete: Leaching Behavior and Ecotoxicity

**DOI:** 10.3390/ma14164476

**Published:** 2021-08-10

**Authors:** Diana Mariaková, Klára Anna Mocová, Kristina Fořtová, Tereza Pavlů, Petr Hájek

**Affiliations:** 1 Research Team Architecture and the Environment, University Centre for Energy Efficient Buildings of Czech Technical University in Prague, Trinecka 1024, 273 43 Bustehrad, Czech Republic; kristina.fortova@cvut.cz (K.F.); tereza.pavlu@cvut.cz (T.P.); petr.hajek@cvut.cz (P.H.); 2 Department of Environmental Chemistry, Faculty of Environmental Technology, University of Chemistry and Technology, Technicka 5, 166 28 Prague, Czech Republic; klara.bajerova@vscht.cz

**Keywords:** high-performance concrete, waste glass powder, leachate, ecotoxicity

## Abstract

This paper deals with the possibility of using different types of waste glass powder in high-performance concrete (HPC) mixtures as a fine fraction replacement. Subsequently, both fractions are used in this research in concrete as a substitute for fine sand and silica flour. To use waste glass in a basic building material such as concrete, it is necessary to verify the basic chemical properties of the selected waste materials. Apart from the basic chemical properties, its environmental impact also appears to be an essential property of waste materials in general. Therefore, the research is mainly focused on the leaching and ecotoxicity experiments on high-performance concrete. HPC mixtures are designed based on the results of the analyzed chemical properties and previous research performed by our research team. Ecotoxicity of these concretes is then verified using Czech standards to evaluate. The results showed a positive impact on the ecotoxic properties of waste glass when used in concrete. A new ecotoxicity classification of waste materials and concrete mixes containing waste materials is proposed as a result of this research and summarized in the conclusion of this paper.

## 1. Introduction

In the last few years, one of the main problems has been the declining number of non-renewable resources and raw materials. However, these materials are closely linked to negative environmental impacts, including high primary energy consumption and CO_2_ emissions. This issue is related to the global increase in waste [1]. According to the latest data from the Czech Statistical Office (from 2019), up to 41% of this waste is generated by construction activities (construction, demolition, reconstruction, etc.). In recent years, there has been an effort to reduce the amount of concrete, which represents the larger volume of building materials used, or improve its impact on the environment.

About 10.9 thousand tons of this waste is glass, and about 39.7 tons of waste glass is from municipal waste [1]. Recycling is a term generally connected to glass and already has very wide importance. Glass from municipal waste is reusable in the form of glass bottles. As a standard, up to 60% of every new glass bottle is made of recycled glass. This process is repeatable for a limited period, and, after that, due to impurities, the glass is usually landfilled [2]. However, glass recycling does not necessarily mean only the reuse of, e.g., glass bottles for the same purpose repeatedly. Utilization in concrete is suitable because the purity demands are lower and the properties of glass are suitable for concrete composition. Czech Republic has a long tradition in jewelry, decoration, and accessory glass production. Accordingly, another used glass comes from brushing the jewelry, where impurities occur as well, and the landfill problem appears again. Due to the tested chemical properties, landfilling has been shown to be unsuitable due to the negative impacts on soil. There is an effort on so-called reuse of waste in other directions. Therefore, it is possible to use glass in a different way from how it was used before [3]. This research deals with the glass component of the photovoltaic panels, which is the last investigated material. Photovoltaic panels are creating an established system for renewable energy utilization. The average lifetime of crystalline silicon photovoltaic module is 25–30 years [4]. This means that the first generation of photovoltaic panel system is coming to the end of its lifetime. Due to the special chemical composition (i.e., high level of aluminum) and higher melting point, landfilling is an inappropriate approach; thus, it is necessary to develop the right way to recycle this technology [5].

Within these intentions, it is appropriate to consider regular and high-quality concretes, which, with lower material consumption, may have the same (or better) properties than regular concrete. This study seeks to provide the possibility that the use of waste glass would bring. It is necessary to be aware of the risks associated with the use of waste materials and to verify, in particular, the impact on the environment and toxicity, given that efforts are being made to reduce the use of primary raw materials not only because their resources are depleted but also due to environmental threats.

Focused on the ingredients of concrete, and especially its effect on the environment, the attention of researchers is preferentially directed towards cement. Its properties when used in concrete are indispensable; however, the energy expended in its processing, in relation to carbon dioxide that is produced, is significant. In terms of CO_2_ emissions, cement has the largest footprint and energy consumption. Therefore, the reduction in size of the cement industry is one of the global sustainability concerns of the 21st century [6]. As a partial replacement for cement, various materials began to be used in our country it—primarily silica flour and microsilica. This paper deals with the mixtures in which silica flour is fully replaced by waste glass powder, and in which microsilica is completely omitted.

Silica flour is formed after the crushing and subsequent grinding of quartz sand to the required roughness. Quality and especially volume uniformity is ensured by modern equipment in production. The properties of silica flour have been investigated in several studies, usually compared with cement and limestone powder as well [7,8]. It has been verified that silica flour produces paste with different rheological properties when substituted at the same volume level [9].

Microsilica (also silica fume) is known as a by-product of the smelting process of silicon metal and ferrosilicon manufacturing. After some research [10,11,12], microsilica was verified as a possible partial replacement for cement in concrete. The knowledge gained about microsilica is partly summarized in a review by Siddique [13]. The paper presents a summary of the physical and chemical properties of microsilica and the reaction mechanism and hardening properties of concrete.

While the use of microsilica in concrete was based on research [10,13,14,15] moving in the right direction, with the increasing consumption of this material, the amount produced as a by-product is not sufficient for use in concrete and microsilica began to be produced on purpose. However, this knowledge returns to the inequality and unecology of this solution and goes back to the core of the problem; it is necessary to look for a material that is purely waste material and has properties that are in some way unsuitable for landfilling and at the same time have similar properties to silica flour, silica fume, or cement, which are originally replaced. The chemical composition is one of the main problems when replacing cement in concrete. There are several types of fume whose chemical composition varies according to the type of produced metal. For example, the fume from a ferrosilicon furnace generally contains more iron and magnesium oxides than from a furnace producing silicon metal [15].

After summarizing all these factors, a convenient raw material for the partial replacement of cement, silica flour, or fine sand in concrete, another perfect pozzolan—glass—was chosen. This choice was based mainly on chemical characterization, which was partly solved in previous research [16]. Regarding the strict limitations and increasing demands of these materials, this research is focused on the problem, which was founded in the idea of replacing cement with silica powder.

The question is whether it is necessary to replace raw materials in concrete. Three types of waste glass powder are used as a full silica flour replacement in concrete. Although the chemical properties vary while using different types of waste glass powder, the properties verified for proper concrete utilization are tested, optimized, and verified [16,17]. Based on the chemical properties, microsilica was fully omitted from one of the mixtures.

To verify the suitability of the use of waste glass and concrete that contains waste glass, specific procedures were chosen according to the decrees of the Czech Republic. One of the main points of this work (apart from the high priority of chemical composition) is ecotoxicity. Ecotoxicology is a multidisciplinary field of modern attitude to deal with the verification of environmental and anthropogenic impacts on ecosystems (aquatic and terrestrial) [18]. The experiments are provided under certain conditions, which are usually given by international organizations such as OECD, ISO, and ASTM in our research [19,20,21,22]. Most of the experiments are included in the European law system.

The ecological risk assessment is mostly expressed in EC50 (effective concentration causing 50% effect in comparison with control). The other possibilities are LC50 (lethal concentration causing 50% effect, in comparison with the control) or LOEC (lowest observed effect concentration) [18]. The values indicate whether the tested samples are hazardous for the environment, and, based on the results, it is possible to evaluate the general ecological danger. The ecological point of view is becoming one of the main factors these days. In the near future, the quality of the environment is gradually becoming the most important thing that will need to be maintained.

The novelty of this study is in the performed ecotoxicology tests, with the focus on building materials such as concrete. It is common that concrete is tested to verify mechanical or physical properties (tensile bending strength, compressive strength, freeze–thaw resistance). However, equally important are the environmental issues and the caused impacts.

The aim of our research was to investigate whether a hazardous glass waste leaching and ecotoxic potential could significantly decrease after incorporation into concrete mix.

## 2. Methodology and Material Characterization

### 2.1. Methodology

**Figure 1:**
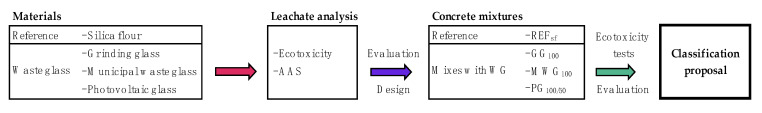
Methodology of experimental work.

The work procedure is graphically explained below in Figure 1.

Three samples of waste glass powders from different sources were chosen. Grinding glass, municipal waste glass, and photovoltaic glass in the form of powder were measured and analyzed to evaluate their impact on the environment. Silica flour (used as the reference sample) was exposed to the same experiments for comparison. AAS was made to collect the basic properties and evaluate the potential risks.

Given the results, the design of concrete mixtures was made. One reference mix as the sample (REF_SF_) and three concrete mixtures containing waste glass (GG_100_, MWG_100_, and PG_100/50_) and ecotoxicity experiments were made to verify the prediction of eliminating the ecotoxicity of waste glass when used in concrete.

After the evaluation of all collected results, our research team came with a methodology. Due to the lack of methodology in this field, the ecotoxicity classification proposal of waste materials and concrete was made.

### 2.2. Material Characterization

Glass is an amorphous pozzolanic material. The properties of different types of waste glass have been examined and tested in previous research [3,23]. Due to the examined properties, such as particle size distribution, chemical analyses, durability, and ecotoxicity experiments, the suitability of waste glass as an aggregate in concrete is explored. This statement is supported by numerous studies [24,25,26,27].

**Figure 2:**
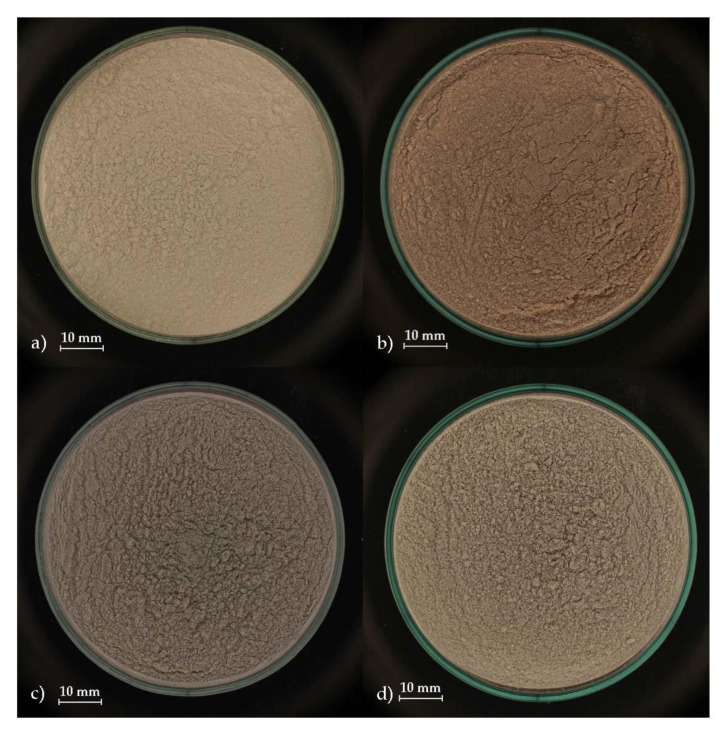
Tested samples: (**a**) SF; (**b**) GG; (**c**) MWG; (**d**) PG.

Three types of waste glass were used in this research and were selected based on the need to recycle them. All samples were tested in the form of powder. A summary of the tested samples is in Table 1 and shown in Figure 2a–d.

Glass from municipal waste is reusable in glass bottles, as mentioned before. This process can be repeated many times until the glass is filled with impurities, and another method of reuse is required. The utilization of glass in concrete is suitable because the purity demands are lower and the properties of glass are suitable for concrete composition.

Another source of used glass is in brushing jewelry. This type of glass powder also contains impurities, but there is a potential for use in concrete due to the pozzolanic properties. The finest fraction is used as a silica flour replacement to create high-performance concrete and verify the chemical properties and microscopic structures.

The last type of glass we explore in this research is glass from photovoltaic panels. Photovoltaic panels are a modern source of energy in the Czech Republic, and their use on a massive scale began approximately 15–20 years ago, which subsequently contributed to the reduction in panel prices in 2009–2010 [28,29]. As the aim of using energy from photovoltaic panels was to reduce CO_2_ emissions, it is appropriate to consider their recycling so that their landfilling does not endanger the soil, for example. In the case of photovoltaic panels, recycling is ideal by disassembling the panels and using them individually. Glass from the photovoltaic panels was crushed into two fractions—fine sand and glass powder (flour). Subsequently, both fractions are used in this research in concrete as a substitute for fine sand and silica flour. The main aims are to save the number of primary resources and eliminate carbon dioxide production [6].

### 2.3. Concrete Mixes

Based on the results of the chemical properties, the concrete mixes were designed. With regard to the results, one reference concrete mix and three concrete mixes containing different types of waste glass were tested.

The reference concrete mixture was made according to the recipe verified by the Department of Architectural Engineering at the Faculty at Civil Engineering, CTU Prague.

The mixture containing waste glass from grinding jewelry (grinding glass, GG) and crushing municipal waste glass (MWG) was added, as a full replacement for silica flour, to the concrete mixture. The replacement, in both cases, was realized in full weight ratio. The replacement ratio is 100% in both mixes (GG_100_ and MWG_100_).

Based on the chemical results and previous research [3,16,23,30], the concrete mixture containing photovoltaic glass was modified. Due to the specific chemical composition of the photovoltaic glass, which was verified in the recent experiments, the adaptation of the mixture was necessary to allow further testing of this mixture. The specification of the composition of this mixture omits microsilica from this type of concrete. This component caused premature cracking of the specimens during concrete hardening.

Full replacement of silica flour was provided by waste glass powder from photovoltaic panels. In addition, 50% of the natural sand (fraction 01/06) was replaced by photovoltaic glass with the requisite fraction (fine sand 01/06). The replacement ratio was 100% with silica flour and 50% with sand (PG_100/50_).

A more detailed description of the composition of the designed concrete mixes is given below in Table 2.

The summary of basic information about the tested concrete mixes is in Table 3, and the final samples are shown in Figure 2a–d. All tested samples were cubes with dimensions 50 × 50 × 50 mm^3^.

**Figure 3:**
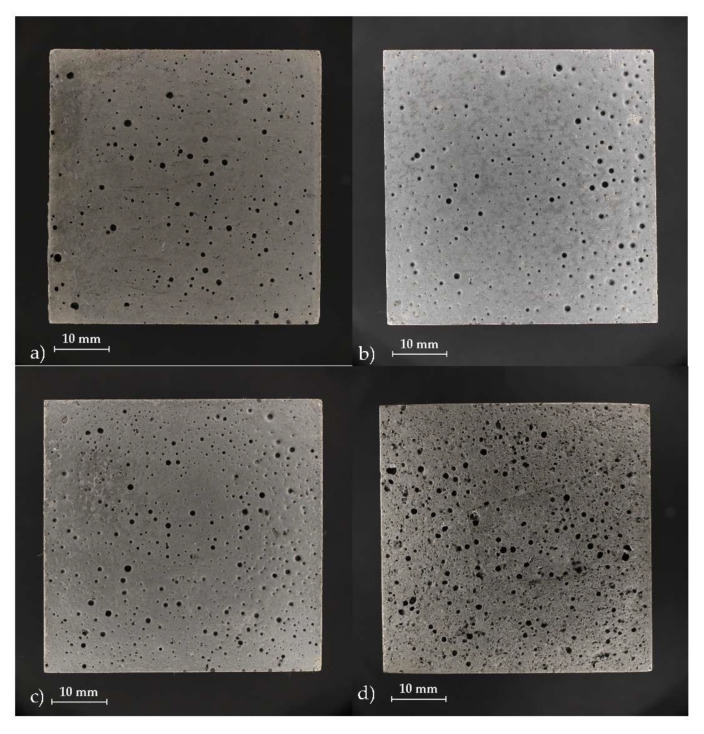
Tested concrete samples: (**a**) REF_SF_; (**b**) GG_100_; (**c**) MWG_100_; (**d**) PG_100/50_.

The concrete samples used for testing ecotoxicology properties are shown in Figure 3. The testing methods of the experiments are described in detail below in Section 2.6 and subsections. The surfaces of the concrete samples differ slightly; this is especially evident when comparing REF_SF_ with PG_100/50_. A more porous structure is a sign of material with lower density, which predetermines different mechanical properties.

### 2.4. Testing Methods

All experiments were made following international standards. The used testing methods are summarized in Table 4. The methodology is described in Section 2.5 and Section 2.6.

### 2.5. Leaching Experiments

For silica flour and glass, air-dried samples of 100 g were mixed with 1000 mL of H_2_O and homogenized on an overhead shaker (7 rpm) for 24 h [19]. Consequently, the solid particles in the leachates were settled for 10 min, and the liquid phase was centrifuged (2360× *g*, 10 min, 25 °C) and filtered through a membrane paper with pores of 5 μm. For the concrete samples, the leachate procedure was adjusted: concrete cubes (aged 28 days) were placed in 3.6 L bottles and covered with H_2_O in the ratio 100 g/1000 mL. The bottles were covered, and the samples were left, without shaking, at room temperature. After 24 h, the concrete cubes were removed, and the leachates were filtrated without centrifugation step. pH and electrical conductivity were determined in the filtrated leachates at room temperature. All leachates were prepared in two replicates. Selected elements (B, Na, Mg, Al, Si, K, Ca, Cr, Fe, Ni, Cu, Zn, As, Se, Mo, Cd, Sb, Ba, Hg, and Pb) were determined using atomic absorption spectrometry with flame atomizer 280FS AA, developed by Agilent Technologies, Inc. (Santa Clara, CA, USA), in leachates after acidification by HCl to a pH of 2.0.

### 2.6. Ecotoxicity Experiments

Ecotoxicological bioassays were performed with original untreated leachates, leachates diluted with nutrient media in a concentration range between 330–800 mL/L, and leachates diluted 10 times and amended with relevant inorganic nutrients according to the control media of the given test species (sample concentration 100 + n mL·L^−1^). pH adjustment was not included in the leachate’s treatment.

#### 2.6.1. Daphnia Acute Toxicity Test

An acute toxicity assay was performed with *Daphnia magna* juveniles aged up to 24 h, which were hatched from ephippia obtained from Microbiotests Inc. (Mariakerke (Gent), Belgium). The experiment was designed following ISO guideline 6341 [21], with some adjustments. Freshly modified ADaM medium (pH ~ 7.3–7.6) prepared according to [31] was used as the control sample.

Five juvenile individuals were transferred into 25 mL beakers with 20 mL of leachate or control sample, covered with transparent film, and put under stable temperature (20 ± 1 °C) and light cycle (fluorescent light, 1000–2000 lx; 16 h light/8 h dark). Each sample was represented by four replicates, whereas the control was represented by six replicates. The inhibition of daphnia mobility (viability) was observed after the 48 h exposition.

#### 2.6.2. Freshwater Algae Toxicity Test

Algae growth inhibition test was performed with the freshwater green algae *Desmodesmus subspicatus*, strain Brinkmann 1953/SAG 86.81, which was obtained from CCALA IBOT, AS CR (Trebon, Czech Republic) partly following the ISO guideline 8692 [22]. Bold Basal Medium (BBM; pH 6.6 ± 0.2) according to [32] was used as the control medium. For the test, 25 mL Erlenmeyer flasks were filled with 15 mL of leachate/control sample and inoculated with precultivated algae (80,000 cells per 1 mL). Samples and controls were represented by triplicates or quadruplicates, respectively. Flasks were covered with sterile cellulose caps and placed under a stable temperature (23 ± 1 °C) and light cycle (16 h of light period) with continuous shaking (130 rpm) for 72 h. An LED light with selected wavelengths (450–455 nm and 660–665 nm) and an illuminance of 3000–3500 lx was used as the light source. Algal cell density was determined via cell counting using a microscope and Bürker chamber (Hecht, Sondheim, Germany). Biomass was determined indirectly as optical density at 684 nm using spectrophotometer Shimadzu UV-1900 (Kyoto, Japan).

#### 2.6.3. Duckweed Growth Inhibition Test

A duckweed assay was proposed by ISO guideline 20079 [20] using *Lemna minor*, strain Steinberg originated from Federal Environmental Agency (Berlin, Germany). Steinberg medium modified by Altenburg (pH 5.5 ± 0.2) [20] served as the control. The test was carried out in 150 mL beakers, filled with 100 mL of sample/control medium. Samples and controls were represented by three and five replicates, respectively. Each vessel was inoculated with 10 fronds of duckweed of a similar total frond area and covered with transparent film. Test vessels were kept in a stable temperature (24 ± 2 °C) and exposed to a light cycle (fluorescent light, 5000–6000 lx; 16 h light/8 h dark).

The total frond area was determined by image analysis using NIS Elements (Version 5.20, Laboratory Imaging, Prague, Czech Republic). Growth rate (GR) was calculated from the values based on repeated measurements during the test exposure, i.e., 0th, 3rd, and 7th day. After the 7-day exposition, fronds were extracted by pure methanol (48 h; 4 °C, dark) and the total chlorophyll content was determined spectrophotometrically (Shimadzu UV-1900) according to [33].

#### 2.6.4. Evaluation of Ecotoxicity Data

In algae and duckweed, the growth rate (GR), based on cell number and frond area, respectively, was calculated using Equation (1):

(1) $$r=\frac{\ln X_{t1}-\ln X_{t0}}{t_{1}-t_{0}}$$

where r is the growth rate per day, *X_t_*_0_ is the value of the parameter in *t*_0_ (d), and *Xt*_1_ is the value of the parameter in *t*_1_ (d). [20].

All ecotoxicological data (daphnia viability, algal and frond GR, algal biomass, and chlorophyll content) were consequently expressed as the values of inhibition/stimulation in percentage, where tested organisms in leachate were compared to control organisms using the following equation:

(2) $$I=\frac{X_{c0-}X_{ci}}{X_{c0}}\times 100$$

where *I* is the inhibition/stimulation of growth (%), *X_c_*_0_ is the average value of control, and *X_ci_* is the average value of sample *i.* [20].

EC50 values were calculated from the inhibition data for all ecotoxicity tests using non-linear regression. A one-way ANOVA and Dunnett’s post-hoc test was performed to compare samples with controls at the α level of 0.05. Based on the Dunnett test, the highest sample concentration, which was statistically not different from the control sample and had no stimulation effect at the same time, was chosen as the NOEC value. The statistical analyses were performed using GraphPad Prism software (Version 9.1, GraphPad Software, San Diego, CA, USA). The level of ecotoxicity was finally classified according to Table 5.

## 3. Results and Discussion

### 3.1. Physico-Chemical Properties of the Leachates

The ecotoxicity testing of wastes is required by the Czech legislation. Provided that the material is miscible with water, chemical analysis and aquatic toxicity of the leachate are performed [34]. According to this decree, the concentration of 13 risk metals (B, Cr, Ni, Cu, Zn, As, Sc, Mo, Cd, Sb, Ba, Hg, and Pb) has to be determined. In the present study, all these metals were below the limit given by the decree and, in vast cases, even below the detection limit. The highest content of Cu (0.11 mg·L^−1^) and Zn (0.11 mg·L^−1^) was found in MWG leachate. These metals can be problematic in natural freshwaters. However, *Desmodesmus subspicatus* (previously named *Scenedesmus subspicatus*) was found tolerant to similar contents of these metals [35]. Therefore, it was more interesting to determine the selected elements that represent the major components in the solid materials, i.e., Na, Al, Si, K, Ca, and trace elements that are important microelements not only for photosynthetic activity (Mg, Fe). The results are showed in Table 6.

In leachates prepared from concrete mixes, the majority of 13 risk metals were also below the detection limit, Ca increased while Na and Si decreased, Al decreased below the detection limit, and Mg and Fe decreased or stayed at a similar level when compared to leachates from glass materials. Higher leaching of the selected elements (Si, Na, Al) in glass powder, compared to concrete cubes, was expected due to the higher surface area of the powders. Moreover, concrete mixes are formed of only approximately 10% of glass powders (GG_100_ and MWG_100_) and around 36.5% of photovoltaic glass and photovoltaic sand (PG_100/50_). Elements which are common components of Portland cement, i.e., Ca and K, were leached more intensively from the concrete mixes, with one exception (GG_100_).

The pH of the leachate also often contributes to the toxicity. Duckweed and algae are relatively tolerant to wider changes; duckweed was reported to survive in the pH up to 9.0 [20], and algae are also tolerant to pH fluctuations [36]. On the contrary, daphnia is not so tolerant to pH changes. Silica flour had a neutral pH, while all glass samples ranged between 10.3 and 10.9, and the concrete mixes had even more alkaline pH values (Table 7). The highest pH value (11.4) was found in PG_100/50_ leachate, and, even after dilution, the pH value remained at a similar level. This might be caused by the highest content of calcium (54.22 mg·L^−1^) in this leachate. The concrete mix with photovoltaic glass had different features from the other concrete mixes, as expected. Due to a high porosity of PG_100/50_ (Figure 3d), the leaching potential was also higher than the rest of the concretes (Table 7). The alkaline pH of concrete leachates decreases after dilution with neutral or acidic water, and the decrease is easier when electrical conductivity is relatively low [37], which was observed for all concrete mixes in this study. The lower conductivity of the leachates, indicating decreased leachability, was most possibly caused by the relatively low surface area of the concrete cubes, in comparison with the homogenized samples [30,37], as well as with the glass materials GG and MWG.

### 3.2. Ecotoxicity Characterization

**Figure 4:**
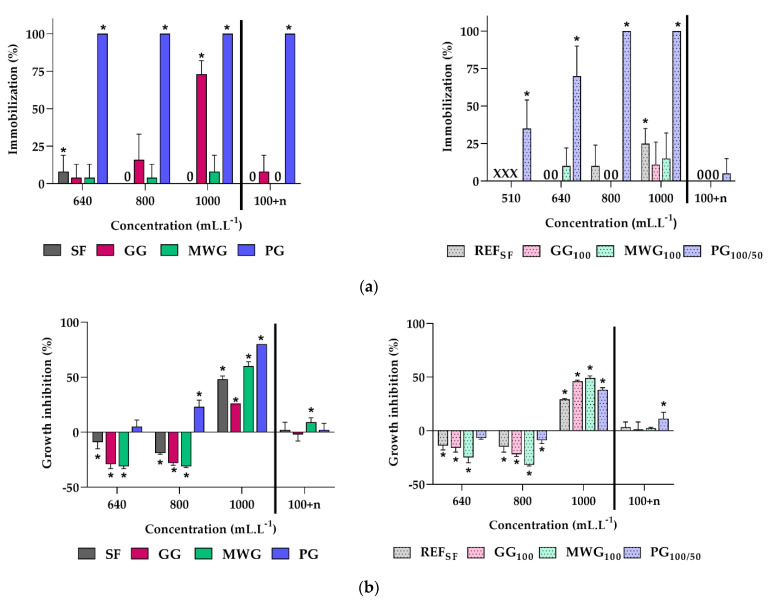

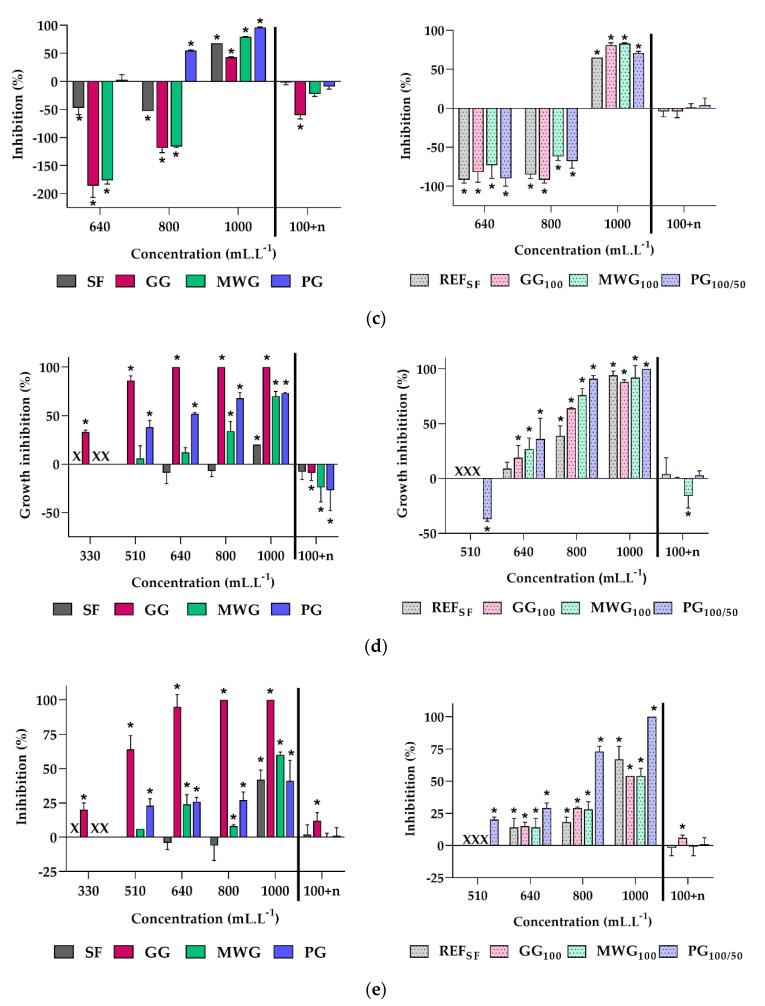
The results of ecotoxicity experiments: (**a**) Daphnia immobilization, (**b**) algal growth rate, (**c**) algal biomass, (**d**) duckweed growth rate, (**e**) duckweed chlorophyll content. X—not determined, 0—zero values, 100 + n—leachates (100 mL·L^−1^) amended with nutrients, *—statistically significant difference from zero values (Dunnett test; α = 0.05).

**Figure 5:**
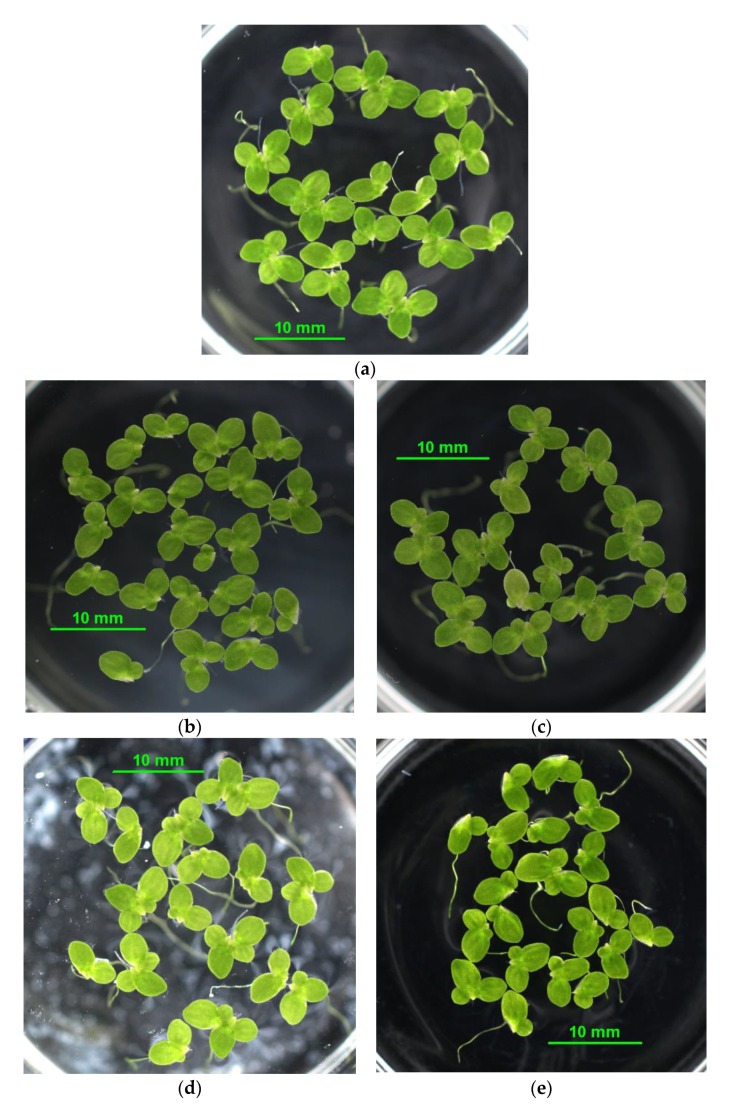

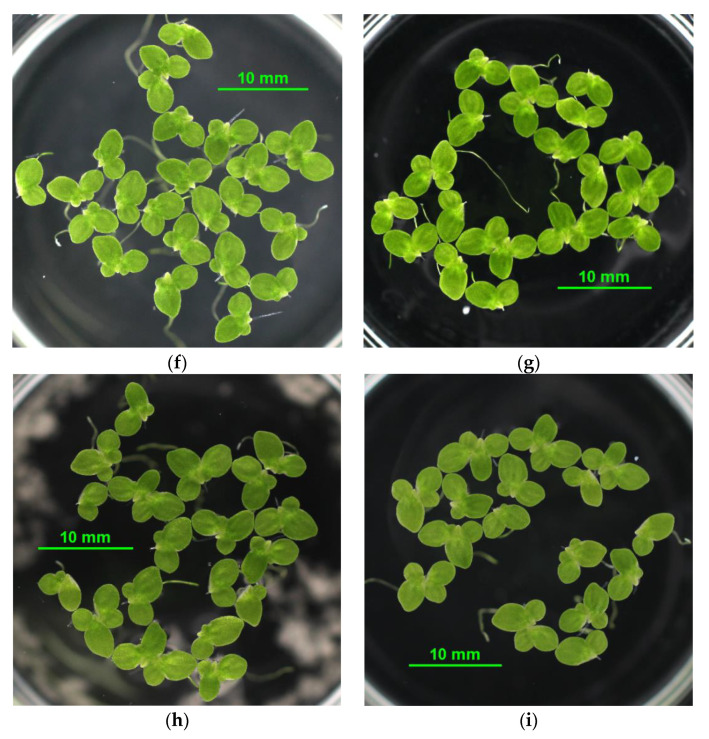
Duckweed test plants photo-documentation: (**a**) control, (**b**) SF, (**c**) REF_SF_, (**d**) GG, (**e**) GG_100_, (**f**) MWG, (**g**) MWG_100_, (**h**) PG, (**i**) PG_100/50_. All samples represent treatment 100 + n (100 mL·L^−1^ with nutrients addition).

The concentrations of the leachates exposed to test organisms were selected in our previous work [30]. The original leachates of both glass materials and concrete mixes usually caused lethality or a high degree of growth inhibition (Figure 4), most likely due to a lack of nutrients. However, the addition of nutrients to concentrated samples was found to be relatively problematic. When nutrients were added to leachates rich in various chemicals, salt precipitation often occurred. Precipitation can cause cluster formation in algae and interfere the precise observation of small organisms such as daphnia, interfere with the algal biomass when optical density is measured, and, in the end, may decrease the nutrient bioavailability. In addition, under natural conditions, the dilution of waste leachate by natural water is a common process, as opposed to enrichment with other nutrients. Therefore, in this study, only original and diluted leachates were taken into account. Furthermore, leachates diluted 10 times followed by nutrient addition, according to the current Czech legislation [34], were included. These samples (100 + n) contained the same amount of nutrients as the control media but only 10% of the original leachate, which led to precipitation of the salts only in leachates with higher content of metals and generally higher values of conductivity, i.e., GG and MWG (Table 6 and Table 7, and Figure 5d,h).

When dilution of the leachate decreased the lethality/high inhibition thoroughly, a sample was considered non-toxic. For this purpose, the original toxicity scale was proposed, as presented in Table 5. The scale based on two sources concerning the characterization of solid wastes was suggested [34,38]. According to current Czech legislation [34], waste leachates diluted with control media to 100 mL·L^−1^ and amended with control nutrients (represented as 100 + n in this study), which cause ≥50% inhibition effect in any test species, are considered ecotoxic, i.e., the original solid waste is classified as hazardous. This category, i.e., hazardous waste, was retained in the proposed scale for waste characterization, mainly for the needs of applied research in the use of wastes as secondary raw materials in the construction sector.

The reference material, silica flour (SF), was found to be non-toxic since only the lack of nutrients in the original leachate inhibited the growth of organisms. This result was expected, because this material is considered inert [39]. Glass is generally also considered an inert material and, so far, has not behaved as ecotoxic. However, there are various types of glass in terms of chemical composition. Glass waste can contain potentially toxic elements, such as As, Cd, Pb, and Zn, in hazardous amounts [40]. Cathode ray tube funnel glass contains high amounts of Pb, due to which the waste glass is classified as hazardous [41]. Another problematic element is aluminum, which was found to be potentially eluted from glass used in the pharmaceutical sector. The elution of aluminum depends on the pH of the eluate and temperature [42,43]. Moreover, glass powder has an increased surface area, and the leaching of potentially toxic elements should be, therefore, examined. The amount of Cu detected in MWG leachate was more than eight times higher than the EC50 reported for daphnia (0.013 mg·L^−1^) [44]; nevertheless, the untreated leachate was only slightly inhibitory to this test species (Figure 3a). This might result from the different chemical composition of the sample. MWG sample was also classified as non-toxic. The GG sample was found inhibitory due to a significant negative effect on duckweed growth and chlorophyll formation (Figure 3d,e). The inhibition could result from the high content of sodium (195 mg·L^−1^), which was approximately 1000 times higher than that of the Steinberg medium, and lack of magnesium (0.47 mg·L^−1^ in GG vs. 9.87 mg·L^−1^ in Steinberg) at the same time. In addition, GG also contained increased levels of aluminium (7.84 mg·L^−1^). Finally, the PG sample was classified as hazardous waste because it was found to be lethal to daphnia at every concentration tested, including the 100 + n sample treatment. The lethality of PG leachate was caused, most likely, by the high content of Al (27.9 mg·L^−1^). Aluminum poses a high environmental risk for the water flea (*Daphnia magna*) and other species of crustaceans. [44] found that 3.9 mg·L^−1^ represented EC50 in the daphnia acute test. Our results were close to this finding, since the nominal concentration of Al in PG (100 + n) mg·L^−1^ was 2.79 mg·L^−1^. The lethality (100% immobilization) of this PG dilution was possibly caused by the presence of other chemicals and the overall different chemical composition of the leachate, in comparison to pure aluminium chemical (AlCl_3_) dissolved in the control medium [44]. One interesting note is that PG leachate did not have such a drastic effect on plant test species.

Aluminium is known to be toxic under highly acidic and highly alkaline conditions, while, under neutral pH, it tends to form insoluble complexes, which are not bioavailable for organisms [45]. The lower toxicity of PG leachate to algae and duckweed could, therefore, result from the lower pH of BBM and Steinberg media, which might neutralize the leachate and lead to the formation of insoluble and, thus, biounavailable particles (Figure 5h). Moreover, as [46] reported, Al can be biosorbed by the extracellular glycoprotein of algae, which decreases the bioaccumulation and consequently the toxicity for algae. Silica was found to be an efficient Al-binding ligand, which decreases the absorption and bioaccumulation of Al in algae [46]. The different ratio of Al:Si in silica flour and waste glass leachates could be, therefore, another reason for the different toxicity of the original materials. The ratio of Al:Si in leachates of the materials decreased in the following order: PG (1.89) > GG (0.96) > MWG (0.20) > SF (0.03), which was in accordance with the decrease in toxicity of the leachates (Table 8).

In the concrete mixes, generally, both EC50 and NOEC values were relatively high (above 500 mL·L^−1^). PG_100/50_ leachate had the lowest EC50 value in daphnia and duckweed, which indicated the highest inhibition effect among the concrete mixes (Table 9). Nevertheless, the differences among samples were not significant, and all concrete leachates were classified as non-toxic. The growth and/or survival of all test organisms at dilutions 100 + n was, in the majority of cases, statistically not different from the control samples (Figure 3 and Figure 4) and all concrete mixes were classified as safe to the environment.

### 3.3. Utilization of Glass Waste in HPC

The solidification of potentially toxic wastes containing heavy metals in concrete mixes, and their further use as construction materials, appears an efficient and sustainable approach [47], which brings several benefits at once. This process stabilizes the toxic substances within the material. The leachability of such stabilized chemicals from the solid material decreases significantly; therefore, they are not bioavailable for organisms. Solidification is often mentioned in connection with the use of fly ash and bottom ash in concrete mixes as a partial substitute for cement [48,49]. Recently, new options of waste utilization have emerged. Hazardous glass waste from cathode ray tube funnels was found recyclable by utilization in ultra high-performance concrete where Pb in concrete leachate was below the limit values [41]. Our research shows the potential use of various types of glass powder in HPC, including photovoltaic waste glass where toxic aluminum is stabilized, making the material safe for the environment.

Using waste of various sources in terms of secondary raw materials reduces the extraction of primary raw materials and reduces the amount of waste disposed in landfills at the same time. All these aspects contribute to a sustainable and environmentally friendly concept in the construction and development sector.

## 4. Conclusions

The toxicity of materials increased in the following order from hazardous to non-toxic:

PG > GG > MWG ~ SF

After incorporation of the materials into concrete mixes, all samples were classified as non-toxic.

This study serves as preliminary research, which will be followed by more extensive testing. The safe usage of waste glass in high-performance concrete needs to be verified by the prolonged leaching period of the concrete mixes. Since this study deals only with acute and semi-chronic ecotoxicity tests, long-term (chronic) toxicity tests will be also performed.

In general, it can be said that the view to building structures in terms of ecotoxicology is an innovation that is not common in the Czech Republic. A new ecotoxicity classification is suggested, which is designed for building materials. Consideration of ecological impacts should be one of the first tendencies in determining the suitability of the use of concrete in the natural environment. The effort to solve this question, such as testing or developing a new methodology, is becoming essential not only in the Czech Republic. This specific point of view is considered a very new approach.

## Figures and Tables

**Table 1:** Summary of tested samples.

Material	Abbreviation	Figure
Silica flour (reference sample)	SF	Figure 1a
Grinding glass	GG	Figure 1b
Municipal waste glass	MWG	Figure 1c
Photovoltaic glass	PG	Figure 1d

**Table 2:** Concrete mixes composition.

Material (kg)	REF_SF_	GG_100_	MWG_100_	PG_100/50_
Portland cement	650	650	650	650
Silica flour	240	-	-	-
Grinding glass	-	240	-	-
Municipal waste glass	-	-	240	-
Photovoltaic glass	-	-	-	240
Sand	1200	1200	1200	600
Photovoltaic sand	-	-	-	600
Microsilica	175	175	175	-
Water	180	180	180	180
Superplasticizer	30	30	30	30

**Table 3:** Summary of tested concrete mixes.

Concrete Mix	Abbreviation	Figure
Reference concrete mix	REF_SF_	Figure 2a
Concrete with grinding glass	GG_100_	Figure 2b
Municipal waste glass concrete	MWG_100_	Figure 2c
Concrete with photovoltaic glass	PG_100/50_	Figure 2d

**Table 4:** Used testing methods.

Method	Experiment	Sample
EN 12457-4	Leachate	REF, GG, MWG, PG
ISO 8692	Algal toxicity test	REF, GG, MWG, PG + concrete mixes
ISO 6341	Daphnia toxicity test	REF, GG, MWG, PG + concrete mixes
ISO 20079	Duckweed toxicity test	REF, GG, MWG, PG + concrete mixes

**Table 5:** Ecotoxicity classification.

Toxicity Class	Abbreviation	NOEC (mL·L^−1^)	EC50 (mL·L^−1^)
Non-toxic (NT)	NT-0	1000	>1000
	NT-1	800	>800
	NT-2	<800	>500
Inhibitory	I	<500	100–500
Hazardous waste	HW	100 + n leachates cause ≥ 50% inhibition	
Mild toxic	MT	<100	10–100
Toxic	T	<10	1–10
Strong toxic	ST	<<1	<1

**Table 6:** Selected elements determined in leachates (mg·L^−1^).

Type	Sample	Ca	K	Na	Si	Al	Fe	Mg
Reference	SF	3.44	1.89	0.71	24.04	<0.8	~0.03	0.29
Waste glass powder	GG	3.00	110.48	194.67	8.22	7.86	~0.08	0.47
	MWG	5.74	4.47	128.30	14.81	3.02	0.21	0.59
	PG	6.14	0.69	87.05	14.74	27.90	~0.11	0.95
Concrete samples	REF_SF_	20.36	18.64	2.21	~3.0	< 0.8	~0.07	0.20
	GG_100_	20.41	20.77	6.20	~2.3	< 0.8	< 0.03	0.17
	MWG_100_	26.47	15.62	3.57	~2.5	< 0.8	~0.04	0.17
	PG_100/50_	54.22	8.83	6.34	5.215	< 0.8	~0.05	0.39

**Table 7:** Conductivity and pH of tested samples (measured in the laboratory temperature 25°C).

Type	Sample	pH	El. Conductivity (µS·cm^−1^)	Weight of Sample (g)
Reference	SF	6.8 ± 0.1	29 ± 1	-
Waste glass powder	GG	10.9 ± 0	606 ± 1	-
	MWG	10.5 ± 0	1291 ± 13	-
	PG	10.3 ± 0	384 ± 7	-
Concrete samples	REF_SF_	11.0 ± 0	245 ± 5	293.55
	GG_100_	11.0 ± 0.1	317 ± 25	273.84
	MWG_100_	11.1 ± 0	317 ± 4	280.6
	PG_100/50_	11.4 ± 0.1	534 ± 3	162.12

**Table 8:** EC50, NOEC values, and ecotoxicity assessment of leachates of materials. GR—growth rate; Chl—chlorophyll content; TC—toxicity class. EC50 and NOEC values expressed in mL·L^−1^.

Material	Daphnia	Algae GR	Algae Biomass	Duckweed GR	Duckweed Chl	Toxicity Level
SF						
EC50	˃1000	˃1000	˃800	˃1000	˃1000	
NOEC	1000	1000	800	800	800	
TC	NT-0	NT-0	NT-1	NT-1	NT-1	non-toxic
GG						
EC50	939	˃1000	˃1000	488	500	
NOEC	800	800	800	<330	<330	
TC	NT-1	NT-1	NT-1	I	I	inhibitory
MWG						
EC50	˃1000	˃800	˃800	882	1000	
NOEC	1000	800	800	640	510	
TC	NT-0	NT-1	NT-1	NT-2	NT-2	non-toxic
PG						
EC50	<100 + n	905	779	654	˃1000	
NOEC	<100 + n	640	640	<510	<510	
TC	HW	NT-2	NT-2	NT-2	NT-2	hazardous waste

**Table 9:** EC50, NOEC values, and ecotoxicity assessment of leachates of concrete mixes. GR—growth rate; Chl—chlorophyll content; TC—toxicity class. EC50 and NOEC values expressed in mL·L^−1^.

Concrete Mix	Daphnia	Algae GR	Algae Biomass	Duckweed GR	Duckweed Chl	Toxicity Level
REF_SF_						
EC50	˃1000	˃1000	800-1000	855	989	
NOEC	800	800	800	640	<640	
TC	NT-1	NT-1	NT-1	NT-2	NT-2	non-toxic
GG_100_						
EC50	˃1000	˃1000	800-1000	746	853	
NOEC	1000	800	800	<640	<640	
TC	NT-0	NT-1	NT-1	NT-2	NT-2	non-toxic
MWG_100_						
EC50	˃1000	˃1000	800-1000	709	857	
NOEC	1000	800	800	<640	<640	
TC	NT-0	NT-1	NT-1	NT-2	NT-2	non-toxic
PG_100/50_						
EC50	639	˃1000	800-1000	620	762	
NOEC	<640	800	800	<510	<510	
TC	NT-2	NT-1	NT-1	NT-2	NT-2	non-toxic

## Data Availability

Data available in a publicly accessible repository.
